# Bloom syndrome patients and mice display accelerated epigenetic aging

**DOI:** 10.1111/acel.13964

**Published:** 2023-08-18

**Authors:** Jamie Lee, Joshua Zhang, Maeve Flanagan, Julian A. Martinez, Christopher Cunniff, Nicole Kucine, Ake T. Lu, Amin Haghani, Juozas Gordevičius, Steve Horvath, Vivian Y. Chang

**Affiliations:** ^1^ Division of Pediatric Hematology and Oncology UCLA Los Angeles California USA; ^2^ Department of Human Genetics UCLA Los Angeles California USA; ^3^ Department of Pediatrics Weill Cornell Medical College New York New York USA; ^4^ Division of Medical Genetics UCLA Los Angeles California USA; ^5^ Department of Psychiatry UCLA Los Angeles California USA; ^6^ Altos Labs San Diego California USA; ^7^ The Epigenetic Clock Development Foundation Torrance California USA; ^8^ Children's Discovery and Innovation Institute UCLA Los Angeles California USA; ^9^ Jonsson Comprehensive Cancer Center UCLA Los Angeles California USA

**Keywords:** Bloom syndrome, cancer, DNA repair, epigenetic aging

## Abstract

Bloom syndrome (BSyn) is an autosomal recessive disorder caused by variants in the *BLM* gene, which is involved in genome stability. Patients with BSyn present with poor growth, sun sensitivity, mild immunodeficiency, diabetes, and increased risk of cancer, most commonly leukemias. Interestingly, patients with BSyn do not have other signs of premature aging such as early, progressive hair loss and cataracts. We set out to determine epigenetic age in BSyn, which can be a better predictor of health and disease over chronological age. Our results show for the first time that patients with BSyn have evidence of accelerated epigenetic aging across several measures in blood lymphocytes, as compared to carriers. Additionally, homozygous *Blm* mice exhibit accelerated methylation age in multiple tissues, including brain, blood, kidney, heart, and skin, according to the brain methylation clock. Overall, we find that Bloom syndrome is associated with accelerated epigenetic aging effects in multiple tissues and more generally a strong effect on CpG methylation levels.

## Graphical abstract

Bloom syndrome is an autosomal recessive disorder caused by germline variants in a DNA repair gene called BLM. Patients with Bloom Syndrome have increased risk of cancer and variable features of accelerated aging. We demonstrate that Bloom Syndrome patients have increased epigenetic aging compared to carriers and that a mouse model of Bloom also shows features of accelerated epigenetic aging in a clock‐ and tissue‐specific manner.

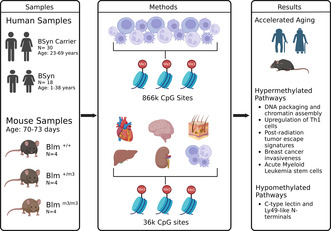

## 1 INTRODUCTION

Bloom syndrome (BSyn) is caused by biallelic null variants in *BLM*, which encodes for a DNA helicase protein in the RecQ family that functions in the maintenance of replication fork stability (Bennett & Keck, 2004; Daley et al., 2014). Cells from patients with BSyn demonstrate a 10‐fold increase in the frequency of sister chromatid exchanges (SCEs) compared to normal patients (Chaganti et al., 1974). Additionally, BLM plays a role in telomere maintenance by interacting with telomere proteins TRF1 and TRF2 (Barefield & Karlseder, 2012; Lillard‐Wetherell et al., 2004; Lu, O'Rourke, et al., 2019; Rezazadeh, 2013). While persistent DNA damage is thought to correlate with aging and drive senescence, it is unresolved whether germline variants in *BLM* cause accelerated aging that can be measured using molecular markers.

Patients with BSyn present with short stature, congenital telangiectatic erythema, and increased susceptibility to infections and cancer, most commonly leukemia, lymphoma, and colorectal cancer (Aktas et al., 2000; Aljarad et al., 2018; Bloom, 1954; Cunniff et al., 2017; Sugranes et al., 2022). Traditional anti‐cancer treatments that cause damage to DNA, such as chemotherapy and radiation, often result in severe life‐threatening toxicities and increased risk of secondary malignancies for patients with BSyn, warranting significant dose modifications from standard practice (Adams et al., 2013; Grasemann et al., 1998; Kataoka et al., 1989). The molecular basis for the early cancers and treatment‐related toxicities seen in patients with Bloom Syndrome is poorly understood beyond the function of BLM in maintaining genomic integrity through its DNA helicase activity.

A different member of the RecQ helicase family of enzymes, WRN, underlies Werner Syndrome (Yu et al., 1996). Werner Syndrome is characterized by signs of premature aging, such as cataracts, early‐onset graying, and skin changes (Chen et al., 2018; de Renty & Ellis, 2017; Takemoto et al., 2013), which are absent in Bloom Syndrome. Yet, cumulative and persistent DNA damage, as seen in BSyn, are thought to contribute to aging (de Winter & Joenje, 2009; Ou & Schumacher, 2018; Ribezzo et al., 2016; Vogel et al., 1999). In fact, it has previously been shown that whole blood from patients with Werner have accelerated epigenetic aging, even after adjusting for chronologic age and blood counts (Maierhofer et al., 2017).

Epigenetic age, an estimator of biological age that can be built from DNA methylation levels, can be a better predictor of health, cancer, and mortality risk than chronological age (Horvath, 2013; Lu, Quach, et al., 2019; Morales Berstein et al., 2022). Epigenetic age acceleration, where the epigenetic age is greater than the chronological age, can occur as the result of segmental progeria such as Down Syndrome (Horvath et al., 2015) and Werner syndrome (Maierhofer et al., 2017). We hypothesized that BSyn patients will have increased epigenetic age due accumulation of DNA damage over time. In this study, we analyzed blood samples from BSyn patients and carriers, as well as a mouse model of Bloom syndrome (Luo et al., 2000), and demonstrate that patients and mice with BSyn have accelerated epigenetic age across multiple measures and tissues.

## 2 RESULTS

### 2.1 Human data

We generated and analyzed data from 18 BSyn samples (age range: 1–38 years) and 30 samples from carriers of BSyn (age range: 23–69 years) (Table 1). They represented different germline variants in *BLM* as well as the Ashkenazi Jewish founder variant noted as *Blm* ^*Ash*^ (German et al., 1977; Li et al., 1998). There were 2 participants with BSyn with unconfirmed variants but had increased sister chromatid exchange and 2 obligate carriers.

**TABLE 1:** Characteristics of patient samples from the Bloom syndrome Registry, Jscreen, and UCLA.

Age at sample collection (years)	Condition	Sex	Variant
0.8	BSyn	M	*BLM* ^*ash*^; *BLM* ^*ash*^
1.8	BSyn	F	275delA; 275delA
1.9	BSyn	F	1933C > T; 3261delT
2	BSyn	F	2695C > T; 3171‐3172insT
2.6	BSyn	M	*BLM* ^*ash*^; *BLM* ^*ash*^
4.3	BSyn	M	*BLM* ^*ash*^; *BLM* ^*ash*^
5	BSyn	M	1642C > T; Deletion of exons 11–12
5.8	BSyn	M	2695C > T; 2695C > T
6	BSyn	M	1642C > T; Deletion of exons 11–12
8.1	BSyn	F	3727insA; 3727insA
16	BSyn	F	*BLM* ^*ash*^; Deletion of exons 3–22
26	BSyn	F	2695C > T; 2695C > T
26.6	BSyn	F	1933C > T; 1933C > T
27	BSyn	F	2695C > T; 2695C > T
32	BSyn	M	*BLM* ^*ash*^ *; BLM* ^*ash*^
36.3	BSyn	M	2506‐2507delAG; 2506‐2507delAG
37	BSyn	M	Unknown
38	BSyn	M	Unknown
23.8	Carrier	F	*BLM* ^*ash*^
24	Carrier	F	Unknown
24.6	Carrier	F	*BLM* ^*ash*^
25.1	Carrier	M	*BLM* ^*ash*^
27.9	Carrier	M	Unknown
29.2	Carrier	F	3261delT
29.7	Carrier	M	275delA
30.8	Carrier	F	275delA
31.4	Carrier	M	2695C > T
31.7	Carrier	M	1933C > T
32	Carrier	M	*BLM* ^*ash*^
32.4	Carrier	F	3727insA
33.8	Carrier	M	*BLM* ^*ash*^
34	Carrier	M	*BLM* ^*ash*^
34.3	Carrier	M	*BLM* ^*ash*^
35.8	Carrier	F	2695C > T
37.8	Carrier	M	3727insA
38	Carrier	M	1642C > T
39	Carrier	F	1642C > T
40	Carrier	M	Deletion of exons 11–12
41	Carrier	M	Deletion of exons 11–12
46	Carrier	F	1933C > T
46.6	Carrier	M	1933C > T
59	Carrier	F	Deletion of exons 3–22
62	Carrier	M	*BLM* ^*ash*^
63	Carrier	M	*BLM* ^*ash*^
64	Carrier	M	*BLM* ^*ash*^
65	Carrier	M	1642C > T
67.5	Carrier	F	2506‐2507delAG
69.4	Carrier	M	2506‐2507delAG

**FIGURE 1:**
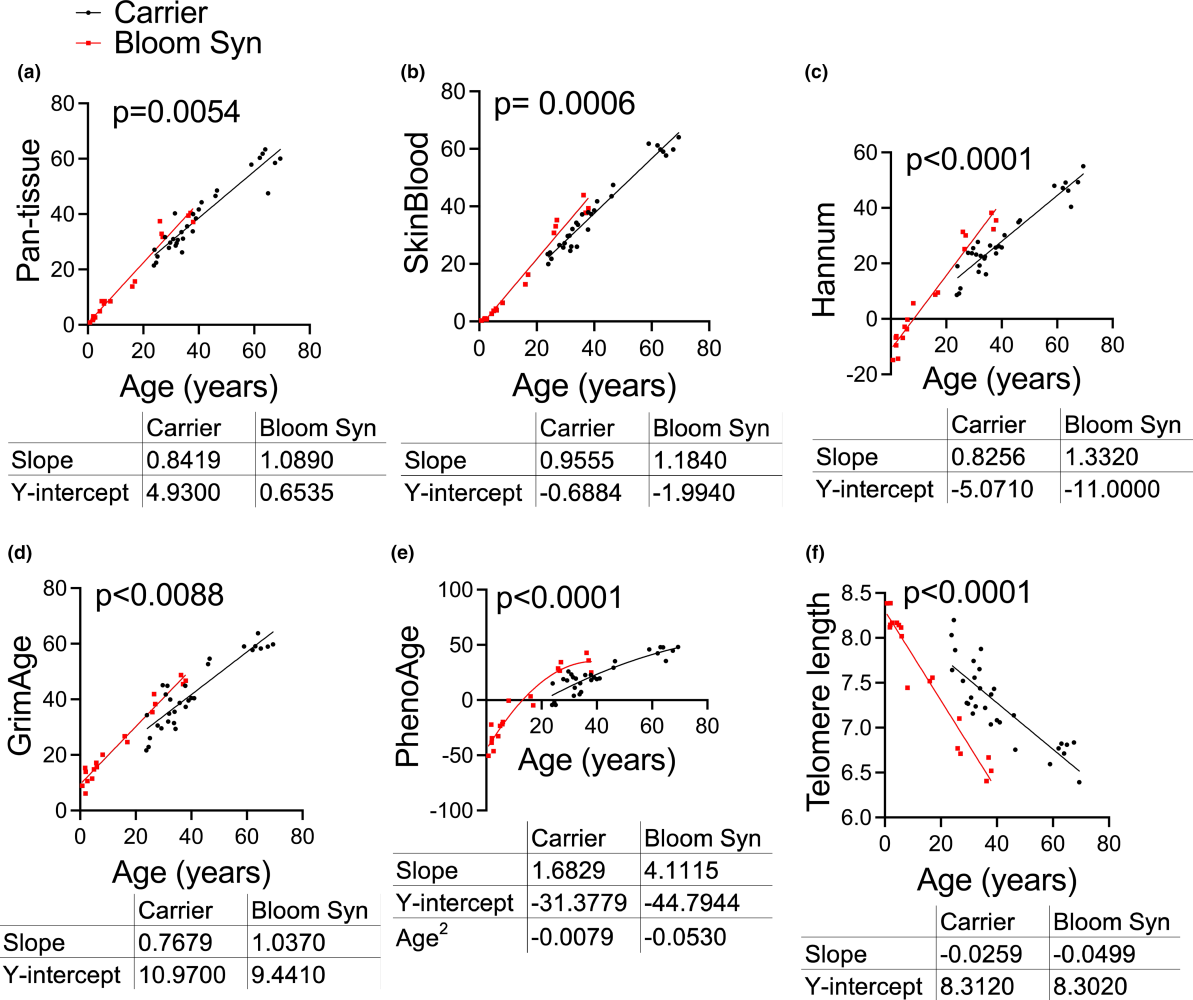
DNA methylation age of BSyn patients and carriers. (a) Pan‐tissue clock, (b) Skin and blood clock, (c) Hannum clock, (d) GrimAge clock, (e) PhenoAge clock, and (f) telomere length. *P*‐values calculated by simple linear regression for pan‐tissue, skin and blood, Hannum, GrimAge clocks and telomere length. Quadratic regression used for PhenoAge.

In our study, we considered several epigenetic clocks that were implemented in the online DNAm methylation (DNAm) age calculator (see Section 4). First, we used the DNA methylation pan‐tissue clock, which is a multi‐tissue predictor of age developed using 8000 samples from 51 healthy tissues and cell types (Horvath, 2013). It quantitatively measures the cumulative effect of aging using 353 CpG sites and applies to a broad spectrum of tissues and cell types. We showed that patients with BSyn have significantly increased DNAm age according to the pan‐tissue clock (Figure 1a).

Although the pan‐tissue clock can be used across all tissues, it does not perform as well when using fibroblasts, which are routinely used for in vitro experiments (Horvath, 2013). The skin and blood clock was subsequently developed to estimate chronologic ages of human fibroblasts, keratinocytes, endothelial cells, skin cells, and other tissues (Horvath et al., 2018). While the original pan‐tissue clock did not detect any age acceleration in Hutchinson Gilford Progeria Syndrome (HGPS), the skin and blood clock applied to fibroblast samples from patients with HGPS did detect accelerated epigenetic age. When we applied the skin and blood clock to our samples, we found that patients with BSyn, like HGPS, have significantly increased DNAm age compared to carriers (Figure 1b).

The Hannum blood‐based clock was developed using methylome data from 656 individuals, ages 19 to 101 years, combined with clinical data such as gender and body mass index (BMI) (Hannum et al., 2013). This model has been shown to apply to other human tissues and detects accelerated aging in tumor tissue. Using Hannum's blood‐based clock, we show that patients with BSyn have increased epigenetic age compared to carriers (Figure 1c).

Patients with BSyn also have an increased GrimAge and PhenoAge, two clocks that predict human lifespan and health span, mortality risk, cancer, physical functioning, and Alzheimer's disease (Figure 1d,e; Horvath, 2013; Levine et al., 2018; Lu, Quach, et al., 2019). Of note, GrimAge is also a DNA methylation‐based estimator of smoking pack years (Lu, Quach, et al., 2019). PhenoAge was trained using clinical measures such as coronary heart disease, physical functioning, familial longevity, cognitive impairment, diet, physical activity, obesity, amongst others, and considered 20,169 CpGs (Levine et al., 2018).

During statistical analyses, we compared linear and quadratic regression fits for age versus each of the epigenetic clocks and found linear fit to be sufficient for pan‐tissue, GrimAge, Hannum, and Skin and Blood clocks, while quadratic fit was better for PhenoAge. Therefore, we used simple linear regression to examine differences between BSyn and carriers for the pan‐tissue, GrimAge, Hannum, and Skin and Blood clocks to compare slopes, and quadratic regression for PhenoAge to compare the coefficient of age squared. The Y‐intercepts for Hannum and PhenoAge are negative values most likely because these epigenetic clocks were trained on adult samples and there are no data on how these clocks perform in children. We also demonstrate that the pan‐tissue, SkinBlood, Hannum, GrimAge, and PhenoAge clocks are highly correlated with each other (Figure S1a,b).

Telomeres are repetitive DNA sequences at the ends of chromosomes that shorten with each cell division (Lopez‐Otin et al., 2013). A specialized DNA polymerase known as telomerase is responsible for preserving the repetitive DNA sequences at telomeres (Zvereva et al., 2010). Senescence or apoptosis is triggered when there is DNA damage at telomeres or when telomeres are critically shortened (Fumagalli et al., 2012; Hayflick & Moorhead, 1961; Hewitt et al., 2012). We used DNA methylation‐based estimates of telomere length (Lu, Seeboth, et al., 2019) and found patients with BSyn had significantly shorter telomere length, DNAmTL, compared to carriers (Figure 1f). DNAmTL only exhibits a weak correlation with actual telomere length (*r* = 0.4), that is, our findings should not be interpreted as statements about actual telomere length. Thus, it is possible that DNAmTL relates to Bloom syndrome while actual telomere length does not. In fact, there are reports of normal telomere length and telomerase activity in BSyn cells (Kaneko et al., 2001). Other studies have shown that BLM functions in alternative lengthening (as opposed to shortening) of telomeres (ALT) independent of telomerase (Acharya et al., 2014; Lu, O'Rourke, et al., 2019; Opresko et al., 2005).

### 2.2 Mouse studies

We then studied epigenetic aging in a Bloom syndrome mouse model created using embryonic stem cell technology (Luo et al., 2000). Mice with null alleles generated using this approach did not develop to term but the *Blm* ^*m3*^ allele gives rise to an aberrant message. *Blm* ^*m3/m3*^ mice are viable, fertile, and have increased sister chromatid exchange, as well as cancer predisposition.

Brain, heart, kidney, liver, skin, and blood were sampled from four wildtype C57Bl6 mice (noted as Blm^+/+^), four heterozygous mice, which were the F1 progeny of wildtype and *Blm* ^*m3/m3*^ mice (noted as Blm^+/m3^), and four homozygous *Blm* ^*m3/m3*^ mice all aged between 70–73 days. There were no statistically significant differences between wildtype and heterozygous Blm^+/m3^ across any of the measured epigenetic clocks (data not shown). Therefore, these groups were combined for downstream analyses.

**FIGURE 2:**
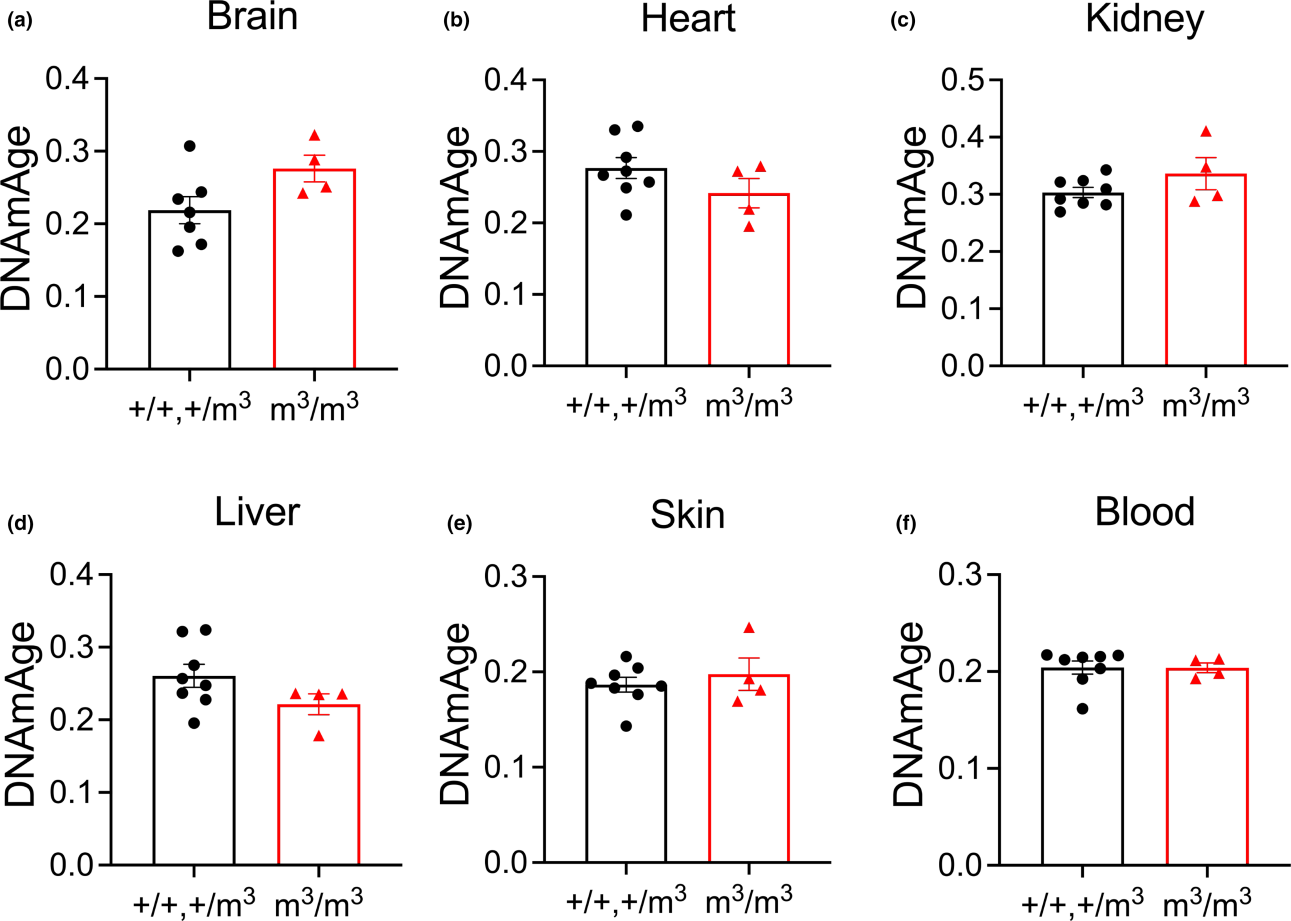
DNA methylation age of mutant Blm mouse model (m^3^/m^3^) compared to wildtype (+/+) and heterozygotes (+/m^3^) using the Pan‐tissue clock. Data are shown as mean ± SEM with comparisons made using unpaired *t*‐test.

**FIGURE 3:**
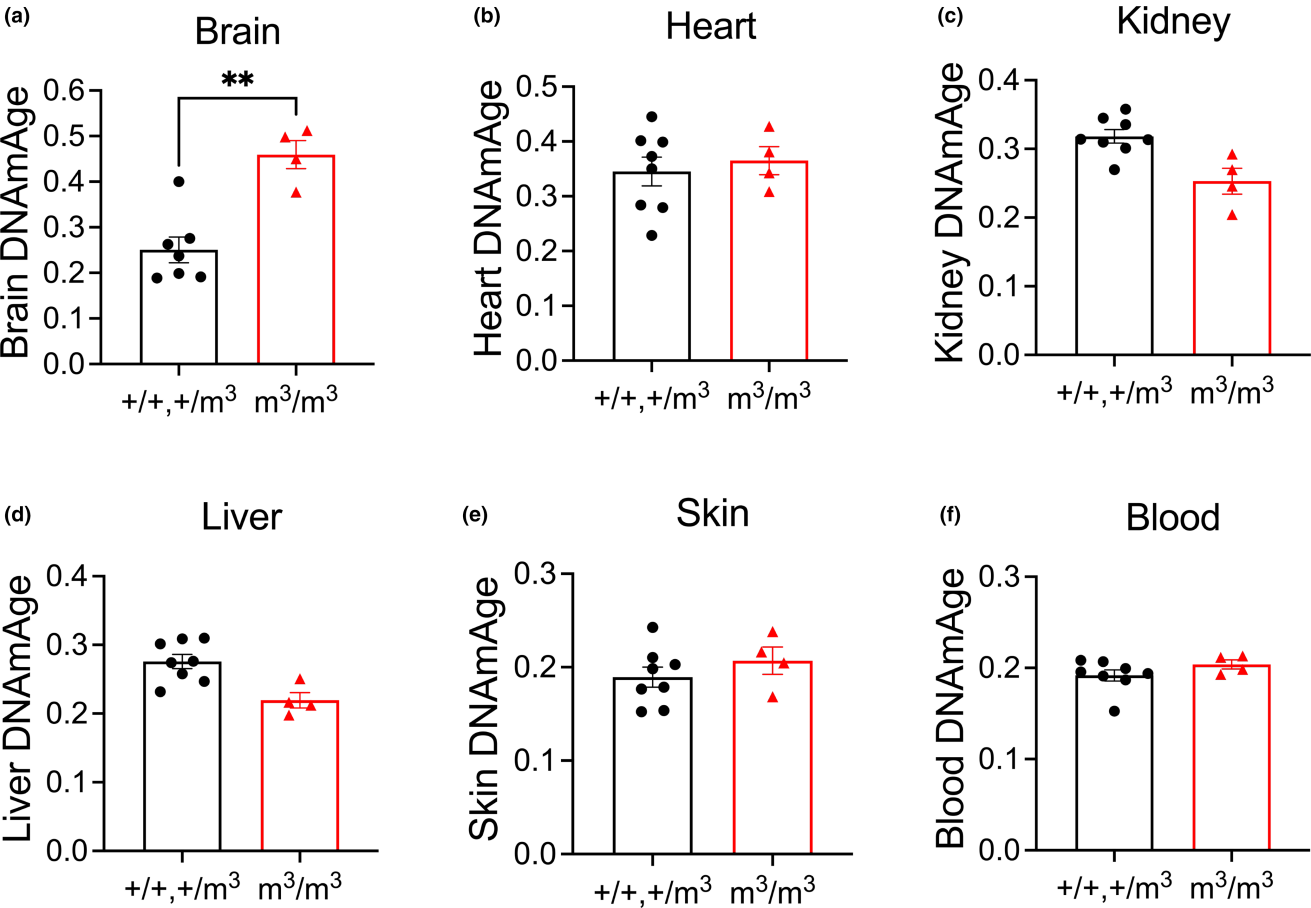
DNA methylation age of mutant Blm mouse model (m^3^/m^3^) compared to wildtype (+/+) and heterozygotes (+/m^3^) using tissue‐specific clocks. Data are shown as mean ± SEM with comparisons made using unpaired *t*‐test. **p < 0.0021.

**FIGURE 4:**
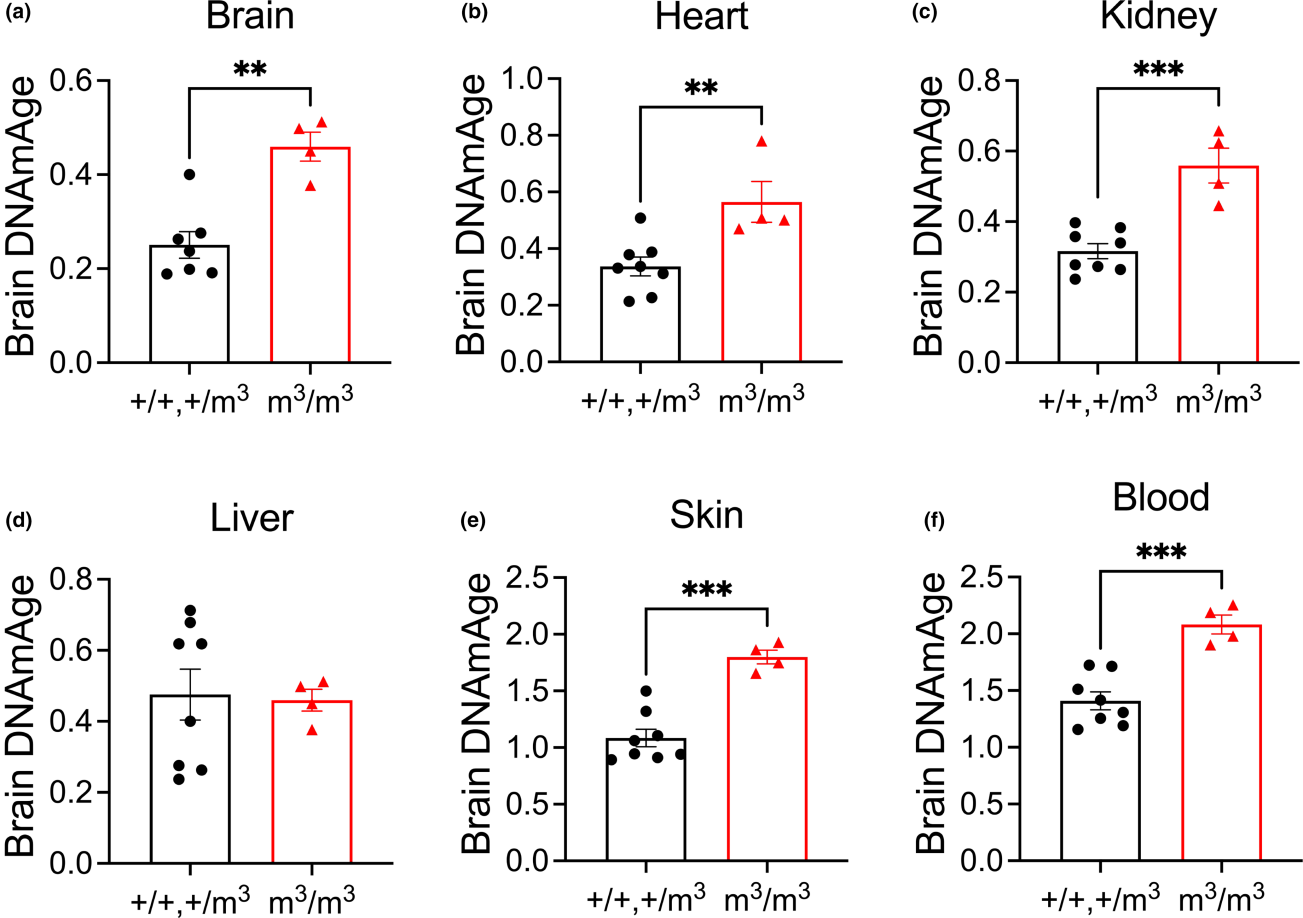
DNA methylation age of mutant Blm mouse model (m^3^/m^3^) compared to wildtype (+/+) and heterozygotes (+/m^3^) using the brain tissue clock. Data are shown as mean ± SEM with comparisons made using unpaired *t*‐test. **p < 0.0021, ***p < 0.0002.

There was no statistically significant increase in DNAm age according to the mouse pan‐tissue clock (Figure 2). The tissue‐specific mouse clocks showed significantly increased methylation aging in brain (Figure 3a) but not heart, kidney, liver, skin, or blood (Figure 3b–f) in *Blm* ^*m3/m3*^ mice compared to wildtype and heterozygous mice. We also found that *Blm* ^*m3/m3*^ mice have increased DNAm age according to the murine brain clock in brain, heart, kidney, skin, and blood (Figure 4a–c,e,f), compared to wildtype and heterozygous mice.

Given the surprising result that the murine brain clock measures increased DNAm in *Blm* ^*m3/m3*^ mice in brain, heart, kidney, skin, and blood compared to wildtype and heterozygous mice, we applied the other tissue‐specific clocks to all other tissues and found that most did not result in any statistically significant differences (data not shown). We created correlation plots using the pan‐tissue and tissue‐specific clocks and were able to demonstrate that the pan‐tissue clock for mouse appears to have limitations in accurately predicting the ages in specific tissues (Figure S2). Furthermore, we demonstrate that the brain clock can apply to heart and kidney tissues but not to skin and liver.

**FIGURE 5:**
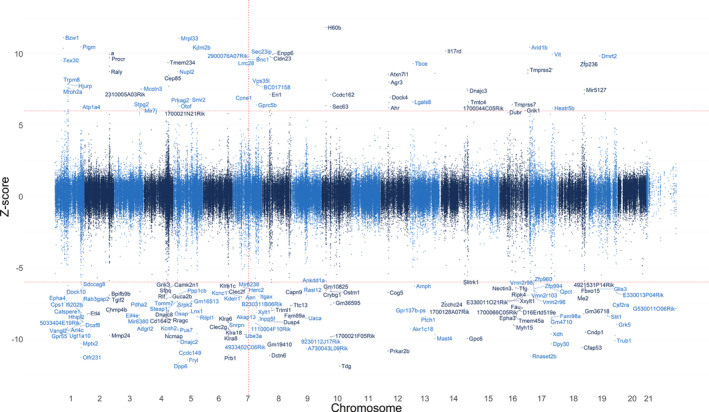
Manhattan plot of median *Z*‐scores from murine epigenome‐wide association study using coded genotypes in a recessive fashion: 0 = WT/ heterozygous, homozygous = 1. The vertical red line indicates location of Blm gene at 7:80454993 using GRCm38/mm10 genome. Horizontal red lines indicate *Z*‐score ± 6.

Epigenome wide association study (EWAS) showed significantly differentially hyper‐ and hypomethylated genes (Figure 5). We used Genomic Regions Enrichment of Annotations Tool (GREAT) to analyze the functional significance of these hyper‐ and hypomethylated regions (McLean et al., 2010). Gene ontology (GO) enrichment analysis showed hypermethylated genes in many biological processes related to DNA conformation and packaging, as well as chromatin assembly, disassembly, and remodeling.

**FIGURE 6:**
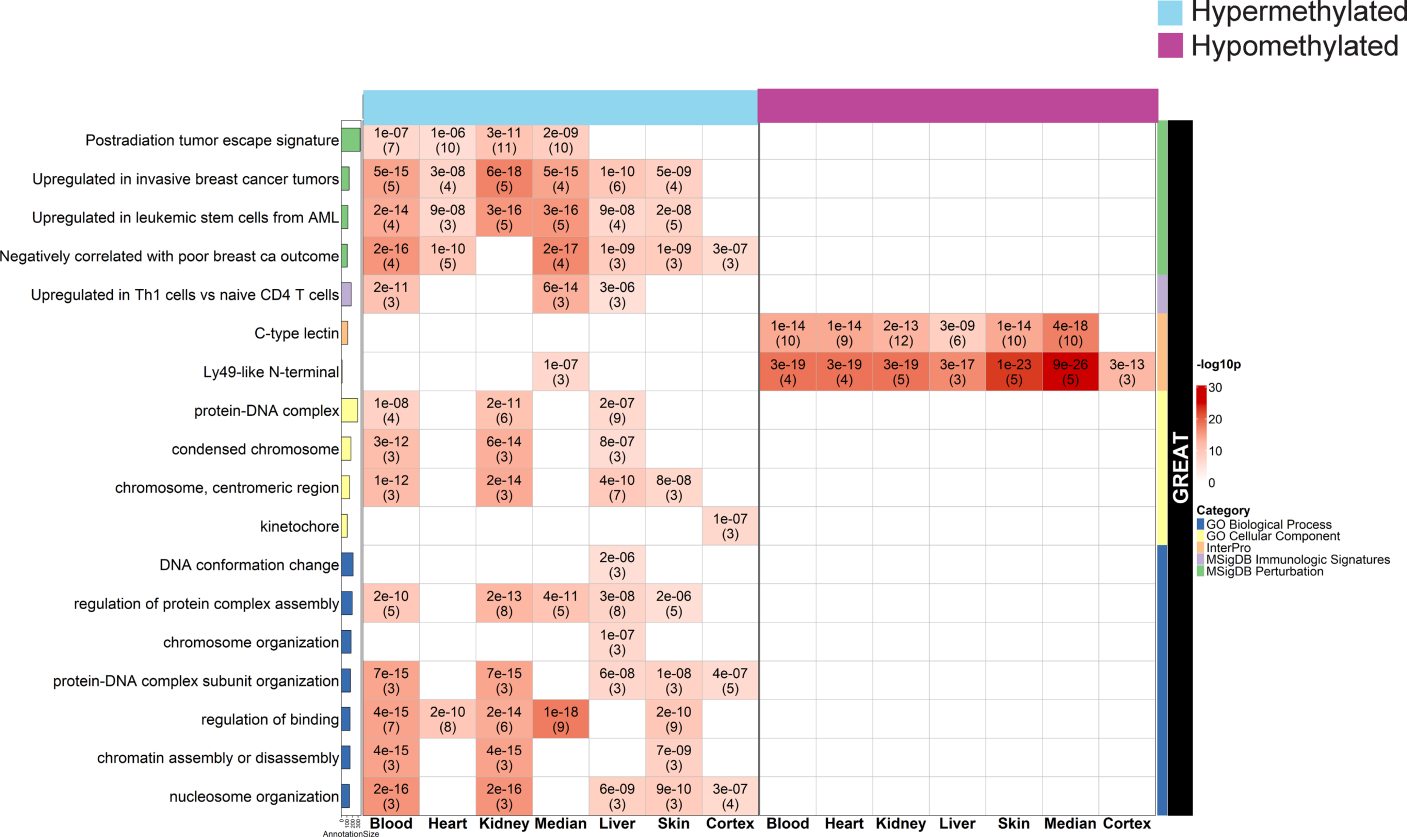
Tissue‐specific GREAT analyses showing annotation of hyper‐ and hypomethylated regions. Bars on the left indicate the number of annotation genes within the custom mammalian array. Each cell presents unadjusted hypergeometric *p*‐values (number of overlapped genes) that have an FDR <0.05 and overlapped genes > = 3 in the heatmap. The heatmap color codes the ‐log10 *p*‐values.

Tissue‐specific GREAT analyses also showed hypermethylated regions in DNA packaging and chromatin assembly, as well as genes involved in upregulation of Th1 cells, post‐radiation tumor escape signatures, breast cancer invasiveness and clinical outcome, and acute myeloid leukemia stem cells (Figure 6; Gu et al., 2014, 2016). Classification of protein families found in hypomethylated regions included C‐type lectin and Ly49‐like N‐terminal, which both function in immunity (Brown et al., 2018; Malarkannan, 2006) and could be related to the mild immunodeficiency seen in some patients with BSyn.

## 3 DISCUSSION

Bloom syndrome is a rare cancer predisposition syndrome thought to be driven by defective DNA repair leading to genomic instability. There is a wide spectrum of clinical phenotypes seen in disorders caused by genomic instability. For example, Hereditary Breast and Ovarian Cancer (HBOC) syndrome caused primarily by variants in *BRCA1/2* which have overlapping functions with *BLM*, is associated with increased cancer risk without syndromic features (Gudmundsdottir & Ashworth, 2006; Wu et al., 2010; Yoshida, 2021). Fanconi Anemia (FA), on the other hand, is a recessive disorder that exhibits increased chromosomal breakage and is associated with growth abnormalities, congenital malformation, bone marrow failure, increased risk of head and neck cancers, and is also increasingly recognized as having early senescence (Helbling‐Leclerc et al., 2021; Kalb et al., 2006; Nalepa & Clapp, 2018). Lastly, a recent study on patients with short telomere syndromes demonstrated that T cell exhaustion rather than genomic instability led to development of solid cancers (Schratz et al., 2023). These examples highlight gaps in our understanding of genomic instability, cancer, and aging.

A recent study investigating the incidence of cancer in individuals from the Bloom Syndrome Registry revealed that 53% were diagnosed with cancer and 35% of individuals with cancer developed multiple cancers (Sugranes et al., 2022). Yet, cancer surveillance in this rare population is challenging due to the variety of cancers that can develop and lack of evidence‐based guidelines (Cunniff et al., 2018; Walsh et al., 2017). Development of a biomarker of aging and cancer would revolutionize clinical care of BSyn as well as many other cancer predisposition syndromes.

Epigenetic clocks have been shown to be robust markers of age and mortality. This study reports, for the first time, a molecular marker of accelerated aging detected in BSyn humans and mice. Interestingly, when we studied different tissues and different epigenetic clocks in the Bloom mouse model, the brain clock most consistently found accelerated aging in the Bloom mice compared to heterozygous and wildtype mice. This may be explained by the decreased proliferative capacity of postnatal mouse brain resembling cellular features from Bloom mice (Semenov, 2021). Indeed, cells from BSyn patients also display decreased DNA replication (Subramanian et al., 2021) and silencing of *BLM* in vitro leads to inhibition of viability and proliferation (Feng et al., 2022). This was a surprising result that merits further investigation.

Carriers of BSyn have not been extensively studied and it is unclear whether they have adverse health impacts (Antczak et al., 2013; Prokofyeva et al., 2013). Studies have found increased risk of colorectal cancer and endometrial cancer in heterozygous *Blm* carriers (Gruber et al., 2002; Schayek et al., 2017) while other studies found no significantly increased prevalence of cancer among carriers (Baris et al., 2007; Cleary et al., 2003; Laitman et al., 2016). This study demonstrates that carriers of BSyn do not appear to have accelerated epigenetic aging compared to BSyn, though we were not able to compare to a population without heterozygous *BLM* variants. Heterozygous mice also had epigenetic age indistinct from wildtype mice.

This study has important limitations for consideration. Due to the rarity of Bloom syndrome and challenges in obtaining precious human samples, there may be confounding variables in the way that samples were obtained, processed, and stored. In addition, we have limited available data on the study participants about other potential confounders such as smoking packyears. It is known that smoking has a strong effect on GrimAge and PhenoAge and though we lack information on this, it is reasonable to assume that most of the Bloom syndrome participants did not have high smoking pack years since they are children or young adults. Lastly, the Bloom mouse model has known limitations because while it does recapitulate important human Bloom syndrome features such as cancer predisposition and radiation sensitivity, the mouse model is hypomorphic and appears to be fertile, unlike patients with Bloom Syndrome.

Future studies are needed to understand whether accelerated epigenetic aging is associated with cancer or other clinical features seen in BSyn and to fully understand the tissue‐specific differences in methylation in better model systems. Pathways identified in our GREAT analyses provide potential mechanisms driving cancer risk in Bloom Syndrome. These findings are expected to lead to the development of improved biomarkers of disease as well as potential therapeutic opportunities.

## 4 METHODS

All study participants were consented on protocols approved by the UCLA and Weill Cornell Institutional Review Boards. Blood samples obtained from the Bloom syndrome Registry were collected, processed, and stored in liquid nitrogen until DNA extraction. All other samples were obtained, processed with DNA extraction within 2 days, and then stored at −80 until sequenced. All animal procedures were performed under protocols approved by UCLA animal care and use committees. All mouse samples were obtained, processed with DNA extraction within 2 days, and then stored at −80 until sequenced.

### 4.1 Methylation analyses

Genomic DNA was extracted from peripheral blood using the PureLink Genomic DNA kit (Thermo Fisher). For human samples, we used the Illumina Infinium Methylation EPIC v2.0 kit (Illumina) and standard Illumina protocols to sequence DNA from BSyn participants and carriers. Briefly, the Illumina bead chip measured bisulfite‐conversion‐based, single‐CpG resolution DNA methylation levels at 866 k CpG sites. The noob normalization method from the minfi R package was used to normalize the human data.

For mouse samples, we used the HorvathMammalMethylChip320 custom mammalian array, which combines a prior custom mammalian array (HorvathMammalMethylChip40) and the 285 k Illumina Mouse Methylation BeadChip (Arneson et al., 2022). The mouse methylation data were normalized using the SeSAMe normalization method (Zhou et al., 2018).

### 4.2 Epigenetic clock software

The human epigenetic clocks were computed using the online epigenetic clock software to calculate the human epigenetic clocks, https://dnamage.genetics.ucla.edu/new. The mouse clocks were trained and developed in different data sets (“DNA Methylation Age Calculator,”). The underlying software code can be found in the supplements of Mozhui et al. (2022).

### 4.3 EWAS

The epigenome‐wide association studies (EWAS) were conducted separately in each tissue type using the *standardScreeningNumericTrait* function from the WGCNA R package (Langfelder & Horvath, 2008). Genotypes were coded as “0” or “1” and used as the trait for the EWAS analysis. For mouse samples, the genotypes were recessively coded as “0” for wildtype and heterozygous mice and “1” for homozygous mice.

### 4.4 GREAT enrichment analysis

The Genomic Regions Enrichment of Annotations Tool (GREAT) (McLean et al., 2010) was used to identify functional annotations of the top BSyn‐related CpGs from the EWAS analyses through the rGREAT R package (Gu et al., 2014, 2016; Gu & Hubschmann, 2022). By using a custom background consisting of the methylation array for each sample, GREAT performed hypergeometric testing using the top EWAS CpGs as foreground and the methylation array appropriate for human or mouse samples as the background. The top 500 CpGs were used for the mouse samples and the top 1000 CpGs were used for the human samples. Annotations were restricted to those with 5 to 3000 gene sets to avoid multiple comparisons. The settings of “Proximal: 5.0 kb upstream, 1.0 kb downstream, plus Distal: up to 50 kb” were used for the enrichment analysis.

### 4.5 Mice

*Blm* ^*m3/m3*^ mice (strain 01BM1) were procured from the NCI Mouse Repository. Heterozygous mice were bred by crossing *Blm* ^*m3/m3*^ and wildtype C57BL/6J mice (strain 000664) from Jackson laboratories. At 70–73 days, male mice were euthanized and whole liver, blood, kidney, heart, skin, and brain tissue were collected for DNA extraction and methylation analyses.

### 4.6 Statistical analyses

Statistical analyses were performed using Prism GraphPad 9. Data are shown as mean+/− SEM. The associations between human methylation data versus age were modeled using linear regression and quadratic regression. Unpaired t‐tests were used to compare Blm mice to wildtype and heterozygous mice.

## AUTHOR CONTRIBUTIONS

JL conducted experiments, sample processing, data analysis, and writing; JZ involved in data analysis and writing. MF, CC, and NK: sample processing, editing. JM involved in data acquisition and editing. AL, AH, and JG involved in data analysis and editing. SH involved in methodology, data analysis, and writing. VYC involved in conceptualization, overall supervision, data acquisition and data interpretation, and writing.

## FUNDING INFORMATION

This work was supported by the National Institutes of Health 1K08HL138305 (VYC).

## CONFLICT OF INTEREST STATEMENT

Steve Horvath is a founder of the non‐profit Epigenetic Clock Development Foundation which plans to license several patents from his former employer (UC Regents). These patents list SH as inventor. The other authors declare no conflicts of interest.

## Supporting information


Figures S1–S2.

## Data Availability

The mammalian methylation array is available from the Epigenetic Clock Development Foundation (https://clockfoundation.org/).
