# Supplementary figures and images for: Bloom syndrome patients and mice display accelerated epigenetic aging
Source: Aging Cell. 2023 Aug 18;22(10):e13964. doi: 10.1111/acel.13964 (PMC10577546; doi:10.1111/acel.13964)

Supplementary Figure 1.

A

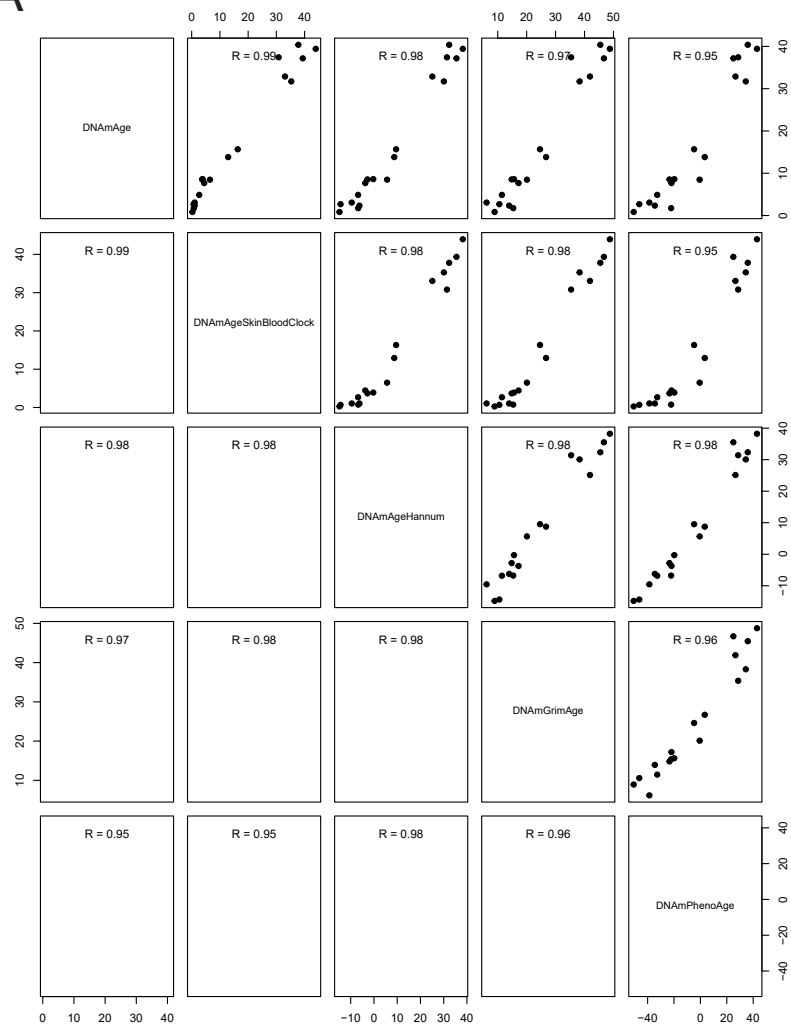

B

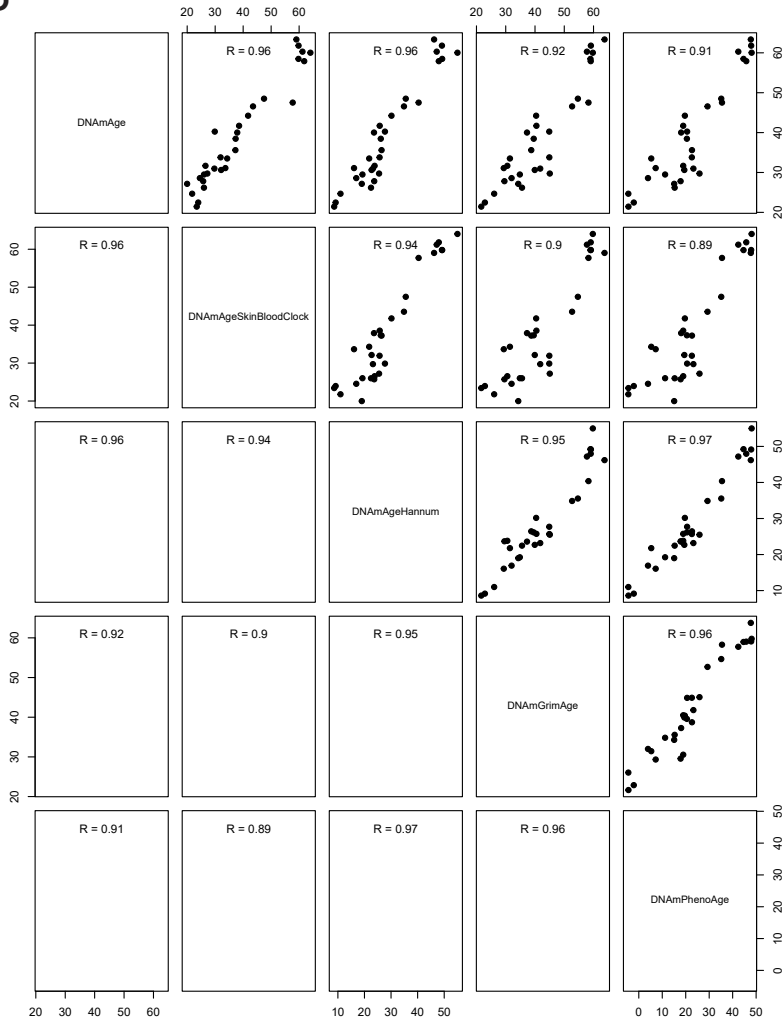

Supplementary Figure 2.

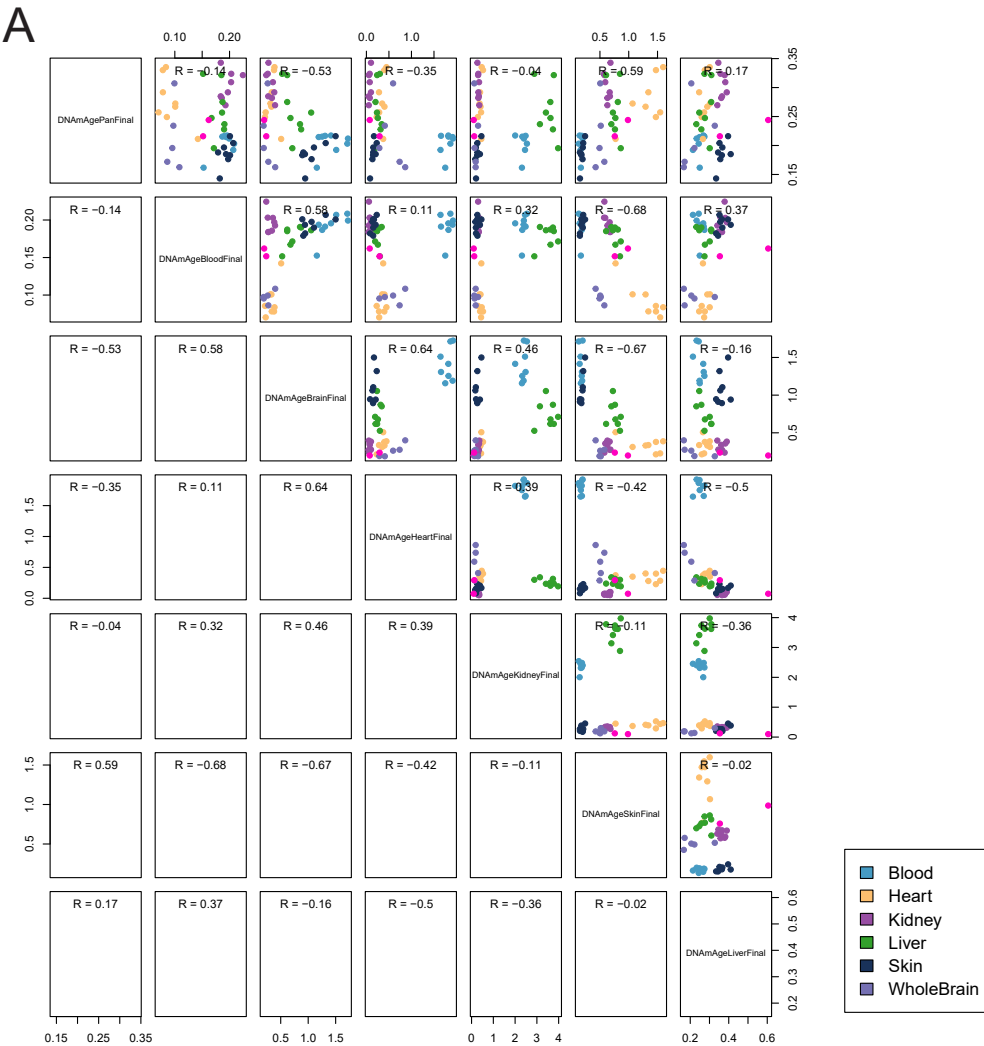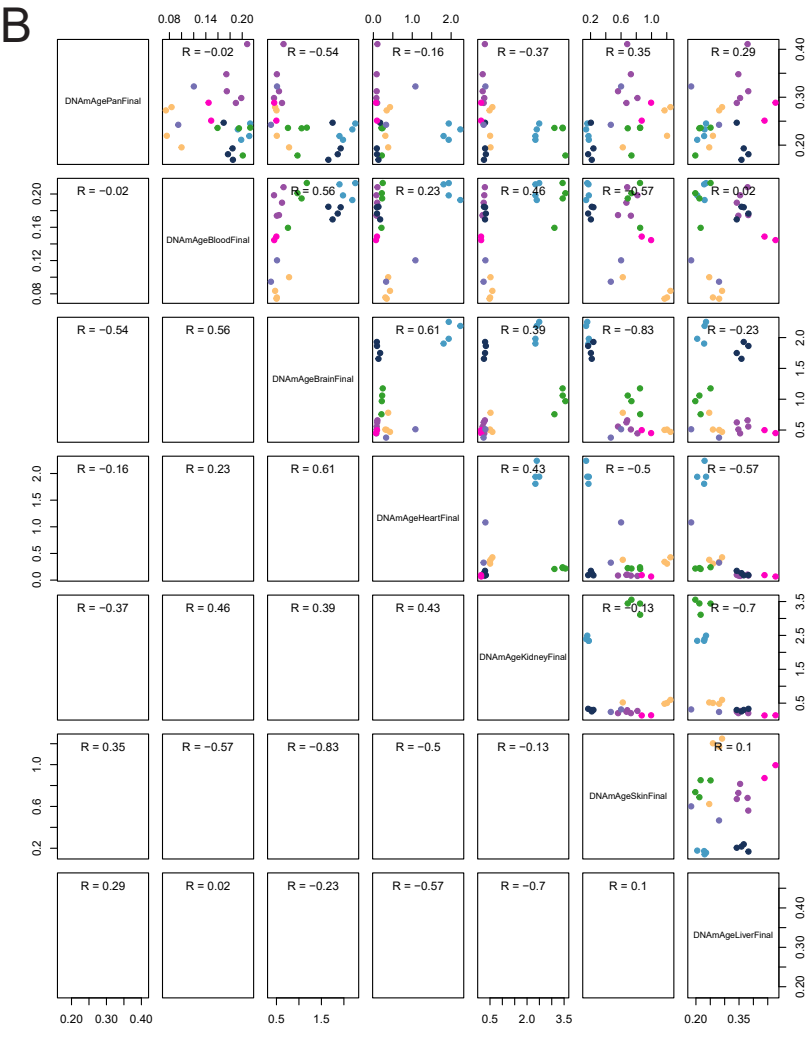

Supplement: Supplementary file 1 — Figures S1–S2. [file ACEL-22-e13964-s001.pdf]
